# Outcomes for acute myocardial infarction with supranormal left ventricular ejection fraction

**DOI:** 10.3389/fcvm.2026.1777247

**Published:** 2026-04-10

**Authors:** Seok Oh, Ju Han Kim, Kyung Hoon Cho, Min Chul Kim, Doo Sun Sim, Young Joon Hong, Youngkeun Ahn, Myung Ho Jeong

**Affiliations:** 1 Department of Cardiology, Chonnam National University Hospital, Gwangju, Republic of Korea; 2 Department of Cardiology, Chonnam National University Medical School, Gwangju, Republic of Korea; 3 Victorian Heart Institute, Monash University, Melbourne, VIC, Australia; 4 Department of Cardiology, Gwangju Veterans Hospital, Gwangju, Republic of Korea

**Keywords:** cardiovascular disease, incidence, left ventricular systolic dysfunction, myocardial infarction, observational study

## Abstract

**Background/aims:**

Despite cumulative evidence of superior outcomes for acute myocardial infarction (AMI) with normal left ventricular ejection fraction (LVEF) compared to those for AMI with reduced LVEF, real-world evidence on outcomes of patients with AMI and supranormal LVEF (snLVEF) is lacking. Therefore, this study aimed to evaluate the clinical outcomes of patients with AMI and snLVEF.

**Methods:**

A total of 27,903 patients with AMI were included from the Korean nationwide AMI cohort between November 2011 and June 2020 after excluding those with unmeasurable LVEF. Patients were classified into four groups according to LVEF: supranormal (≥65%), normal (50%–64%), mid-range (40%–49%), and reduced (<40%). The primary outcome was 3-year all-cause mortality.

**Results:**

Across four hierarchical Cox models, snLVEF was consistently associated with lower 3-year all-cause mortality compared with normal LVEF (a crude model HR 0.71; 95% CI 0.58–0.87; a fully-adjusted model HR 0.77; 95% CI 0.60–0.98), with similar estimates observed in intermediate models.

**Conclusions:**

Patients with AMI and snLVEF experienced the best clinical outcomes with the lowest mortality across the four groups.

## 1 Introduction

Acute myocardial infarction (AMI), a medical emergency that requires timely medication and revascularization, is considered one of the main causes of death and disability worldwide (1). Although the incidence of AMI has declined in the United States and Europe, its incidence continues to increase in many other countries, causing a considerable socioeconomic burden. Since patients with AMI tend to have a higher risk of adverse cardiovascular events, the identification of prognostic factors for AMI is clinically important for secondary prevention.

The left ventricular ejection fraction (LVEF) is a well-established indicator of left ventricular (LV) systolic function and is widely measured in clinical practice. The LVEF is recognized as one of the most potent prognostic factors of mortality in diverse clinical manifestations of coronary artery disease (CAD), including AMI (2). In other words, a low LVEF is responsible for a higher risk of adverse cardiovascular events among these patients (2). Although most studies on the association between LVEF and clinical outcomes have focused on this point, a consensus regarding the association between supranormal LVEF (snLVEF: LVEF ≥65%) and clinical outcomes in these patients has not been reached.

We hypothesized that, contrary to prior reports suggesting a U-shaped association between LVEF and mortality, patients with AMI and snLVEF would not exhibit excess mortality compared with those with normal LVEF.

## 2 Methods

### 2.1 Ethical statement

This study adhered to the principles of the Declaration of Helsinki, and its protocol was approved by the Institutional Review Board (IRB) of Chonnam National University Hospital (IRB No. CNUH-2024-179). The requirement for informed consent was waived due to the retrospective nature of the analysis.

### 2.2 Study population

The Korean Acute Myocardial Infarction Registry (KAMIR) is a South Korean nationwide multicenter observational cohort study of patients with AMI. The KAMIR-National Institutes of Health (KAMIR-NIH) cohort (November 2011 to December 2015) comprised patients with AMI who were treated at 20 major tertiary cardiovascular institutions capable of performing both percutaneous coronary intervention (PCI) and coronary artery bypass grafting (CABG), while the KAMIR-V cohort (January 2016 and June 2020) consisted of patients with AMI who were treated at 33 major tertiary cardiovascular institutions capable of both PCI and CABG. All consecutive patients with AMI admitted to tertiary hospitals in South Korea during these periods were enrolled in these two KAMIR cohorts. The study protocol was approved by the ethics committees of all participating institutions (3).

**Figure 1:**
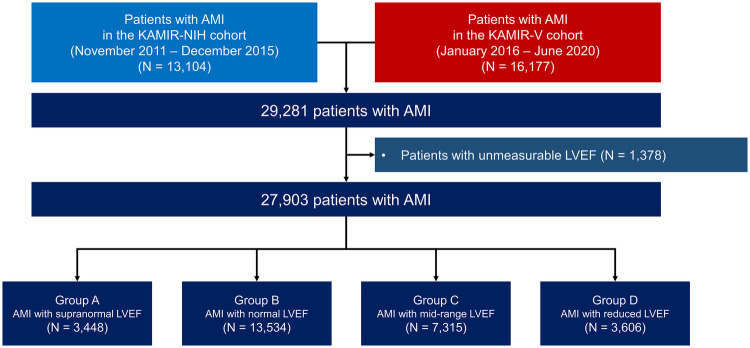
Study flow diagram. AMI, acute myocardial infarction; KAMIR-V, Korea Acute Myocardial Infarction Registry-V; LVEF, left ventricular ejection fraction.

Of the 29,281 patients with AMI, a total of 27,903 patients were included in the final analysis after the exclusion of those with an unmeasurable LVEF (*n* = 1,378). They were subdivided into four groups according to LVEF: Group A, AMI with snLVEF (LVEF ≥65%) (*n* = 3,448); Group B, AMI with normal LVEF (LVEF 50%–64%) (*n* = 13,534); Group C, AMI with mid-range LVEF (LVEF 40%–49%) (*n* = 7,315); and Group D, AMI with reduced LVEF (LVEF <40%) (*n* = 3,606) (Figure 1).

### 2.3 Definitions

Based on international guidelines (4, 5), AMI was defined as myocardial injury evidenced by elevated cardiac biomarkers and at least one of the following conditions: myocardial ischemia-related symptoms or signs, ST-segment changes and/or development of pathological Q-waves on a 12-lead electrocardiogram, novel loss of myocardial viability or abnormalities in regional wall motion on cardiovascular imaging, and evidence of intracoronary thrombus during coronary angiography. ST-segment elevation myocardial infarction (STEMI) was defined as clinical evidence of AMI with novel developmental ST-segment elevation in two or more continuous leads, >0.2 mV in precordial leads V1–V3, or >0.1 mV in all other leads of a 12-lead electrocardiogram.

In the present study, LVEF was measured during the index hospitalization (typically within 24–72 h after PCI) using two-dimensional transthoracic echocardiography across all participating centers in the KAMIR-NIH and KAMIR-V cohorts. Quantitative LVEF assessment was primarily performed using the modified Simpson's biplane method from apical two- and four-chamber views, in accordance with the recommendations of the American Society of Echocardiography (6). In cases where image quality was deemed sufficient, M-mode echocardiography was also utilized.

Angiographic and procedural characteristics were reviewed and recorded. The infarct-related artery (IRA) was defined as the coronary artery liable for the clinical manifestations of AMI, which was occluded or stenosed, with intracoronary atherothrombotic events. The characteristics of the coronary lesions in the IRA were categorized using the American College of Cardiology/American Heart Association (ACC/AHA) lesion classification. Coronary flow of the IRA was graded based on the Thrombolysis in Myocardial Infarction (TIMI) flow grade. Significant stenosis of the coronary artery was defined as ≥50% and ≥70% in lumen narrowing of the left main coronary artery (LMCA) and other epicardial coronary arteries (i.e., left anterior descending coronary artery, left circumflex coronary artery, and right coronary artery), respectively. Multivessel disease was defined as the presence of two or more significant stenoses of the coronary arteries.

All other clinicodemographic profiles of the study participants were assessed and defined in accordance with standardized definitions provided by the committee boards of both the KAMIR-NIH and KAMIR-V cohorts (3).

### 2.4 Clinical outcomes

Clinical follow-up of the study participants was performed for approximately 36 months after enrollment. Follow-up examinations were performed after 6 months, 1 year, 2 years, and 3 years after the intervention via outpatient visits, medical record review, telephone follow-up, or whenever any adverse cardiovascular events occurred. The primary outcome was all-cause death, a composite outcome of cardiac and non-cardiac deaths. The exploratory outcomes included major adverse cardiac and cerebrovascular events (MACCE), cardiac death, non-cardiac death, nonfatal myocardial infarction (NFMI), any revascularization, cerebrovascular accident (CVA), and readmission. MACCE was defined as a composite outcome of all-cause death, NFMI, any revascularization, CVA, and readmission. Any revascularization was defined as any event of repeat PCI of any segment of the coronary lesion or CABG. Readmission was defined as first-time hospitalization owing to other causes such as angina or heart failure (HF).

### 2.5 Statistical analyses

Statistical analyses were performed to compare baseline characteristics and clinical outcomes among the four LVEF groups (Group A–D). All analyses were conducted using SPSS (version 25.0; SPSS Inc., Armonk, NY, USA). Continuous variables are presented as mean ± standard deviation or median with interquartile range, as appropriate, and categorical variables are expressed as frequencies and percentages. Between-group comparisons were performed using analysis of variance, the Kruskal–Wallis test, Student's *t*-test, or the chi-square test, according to variable distribution and data type. A two-sided *P* value <0.05 was considered statistically significant.

Time-to-event outcomes were analyzed using Cox proportional hazards regression models to estimate hazard ratios (HRs) and 95% confidence intervals (CIs) for each LVEF category, using the normal LVEF group (Group B) as the reference. To evaluate the robustness of the association between LVEF category and clinical outcomes, four hierarchical models with incremental adjustment were constructed.

Model 1 was an unadjusted model including only LVEF category. Model 2 was adjusted for age and sex. Model 3 additionally incorporated baseline clinical characteristics, comorbidities, renal function, angiographic findings, and procedural variables. Model 4 (fully adjusted model) further included discharge hemodynamic parameters and guideline-directed medical therapies. Detailed information regarding covariate selection and model construction is provided in Supplementary Data S1.

Covariates were selected *a priori* based on clinical relevance and evidence from prior AMI studies rather than automated selection procedures. The proportional hazards assumption was evaluated using log-minus-log survival plots, and no major deviation from proportionality was observed. Patients with missing data for covariates included in each model were excluded from that specific analysis using a complete-case approach.

To evaluate the robustness of the predefined snLVEF cutoff (≥65%), a sensitivity analysis was performed using an alternative threshold of ≥60%, with parallel categorical Cox regression analyses conducted under the same hierarchical modeling framework. Additionally, LVEF was modeled as a continuous variable using restricted cubic splines to assess potential non-linearity.

## 3 Results

### 3.1 Baseline characteristics

Table 1 summarizes the clinical characteristics of the participants in the four groups. The mean LVEF values were 68.78% (Group A), 56.80% (Group B), 45.24% (Group C), and 31.97% (Group D).

**Table 1:** Baseline characteristics of study participants.

Variable	Group A	Group B	Group C	Group D	*P*-value for trend
	LVEF ≥65%	LVEF 50–64%	LVEF 40–49%	LVEF <40%	
	(*n* = 3,448)	(*n* = 13,534)	(*n* = 7,315)	(*n* = 3,606)	
Age, years	63.01 ± 12.32	62.75 ± 12.28	64.56 ± 12.67	68.30 ± 12.40	<0.001
Age ≥75 years	712 (20.6)	2,665 (19.7)	1,882 (25.7)	1,288 (35.7)	<0.001
Male sex	2,568 (74.5)	10,531 (77.8)	5,477 (74.9)	2,587 (71.7)	<0.001
ODT, h	38.27 ± 229.75	29.19 ± 218.16	26.45 ± 180.45	38.70 ± 200.95	<0.001
DBT, h	16.76 ± 34.23	13.69 ± 33.80	11.23 ± 34.37	19.83 ± 57.98	<0.001
BMI ≥25 kg/m^2^	1,367 (41.9)	4,979 (38.9)	2,384 (34.6)	946 (28.1)	<0.001
Killip class III-IV	178 (5.2)	967 (7.3)	851 (11.8)	1,051 (29.7)	<0.001
Comorbidities					
Hypertension	1,774 (51.5)	6,680 (49.4)	3,595 (49.2)	1,989 (55.2)	0.002
Diabetes mellitus	839 (24.3)	3,494 (25.8)	2,061 (28.2)	1,464 (40.6)	<0.001
Dyslipidemia	502 (14.6)	1,800 (13.3)	878 (12.0)	423 (11.7)	<0.001
Prior CAD	520 (15.1)	1,783 (13.2)	1,062 (14.5)	799 (22.2)	<0.001
Prior CVA	208 (6.0)	772 (5.7)	513 (7.0)	378 (10.5)	<0.001
Smoking history	1,935 (57.7)	7,773 (59.1)	4,053 (56.9)	1,790 (51.5)	<0.001
Family history of CAD	287 (8.6)	1,088 (8.3)	447 (6.3)	205 (5.9)	<0.001
Use of thrombolysis	16 (0.5)	95 (0.7)	92 (1.3)	22 (0.6)	0.017
Multivessel disease	1,484 (43.3)	6,496 (48.3)	3,776 (52.0)	2,278 (64.8)	<0.001
LMCA disease	138 (4.0)	564 (4.2)	343 (4.7)	320 (9.1)	<0.001
Use of PCI	2,973 (86.2)	12,512 (92.5)	6,889 (94.2)	3,187 (88.4)	<0.001
Use of femoral approach	1,322 (44.5)	6,266 (50.1)	3,883 (56.4)	2,001 (62.8)	<0.001
Use of GPIIb/IIIa inhibitors	239 (6.9)	1,632 (12.1)	1,031 (14.1)	376 (10.4)	<0.001
Use of thrombus aspiration	314 (9.1)	2,357 (17.4)	1,508 (20.6)	592 (16.4)	<0.001
Use of intracoronary imaging	833 (24.2)	3,131 (23.1)	1,701 (23.3)	640 (17.7)	<0.001
Infarct-related artery					<0.001
LMCA or LAD	1,351 (45.3)	5,000 (39.9)	4,122 (59.8)	2,095 (65.7)	
LCX or RCA	1,628 (54.7)	7,521 (60.1)	2,770 (40.2)	1,092 (34.3)	
ACC/AHA lesion type B2/C	2,385 (82.0)	10,352 (85.4)	5,729 (86.5)	2,738 (89.0)	<0.001
Completeness of PCI					<0.001
Complete PCI	2,060 (59.7)	8,725 (64.5)	4,415 (60.4)	1,777 (49.3)	
Incomplete PCI	1,388 (40.3)	4,809 (35.5)	2,900 (39.6)	1,829 (50.7)	
TIMI flow grade 0-I	1,242 (42.3)	6,973 (56.4)	4,612 (67.8)	1,942 (62.0)	<0.001
LVEF, %	68.78 ± 3.79	56.80 ± 4.11	45.24 ± 2.75	31.97 ± 6.22	<0.001
eGFR <60 mL/min/1.73m^2^	430 (12.5)	1,800 (13.3)	1,378 (18.9)	1,361 (37.8)	<0.001
STEMI as a final diagnosis	1,031 (29.9)	5,962 (44.0)	4,415 (60.4)	1,879 (52.1)	<0.001
SBP at discharge, mmHg	117.44 ± 15.54	115.82 ± 15.26	112.99 ± 15.18	111.56 ± 16.05	<0.001
DBP at discharge, mmHg	69.96 ± 10.16	69.37 ± 10.17	68.04 ± 10.06	66.70 ± 10.12	<0.001
Heart rate at discharge, bpm	69.13 ± 9.97	70.01 ± 10.32	72.32 ± 10.68	75.23 ± 12.10	<0.001
Medications					
Aspirin	3,411 (98.9)	13,460 (99.5)	7,279 (99.5)	3,557 (98.6)	0.111
P2Y12 inhibitors	3,399 (98.6)	13,423 (99.2)	7,258 (99.2)	3,547 (98.4)	0.250
Beta-blockers	2,565 (74.4)	10,803 (79.8)	5,951 (81.4)	2,565 (71.1)	0.004
RAAS inhibitors	2,613 (75.8)	10,329 (76.3)	5,524 (75.5)	2,450 (67.9)	<0.001
Statins	3,252 (94.3)	12,795 (94.5)	6,829 (93.4)	3,046 (84.5)	<0.001
In-hospital death	21 (0.6)	89 (0.7)	124 (1.7)	299 (8.3)	<0.001

ACC/AHA, American College of Cardiology/American Heart Association; BMI, body mass index; CAD, coronary artery disease; CVA, cerebrovascular accident; DBP, diastolic blood pressure; DBT, door-to-balloon time; eGFR, estimated glomerular filtration rate; GPIIb/IIIa, glycoprotein IIb/IIIa; LAD, left anterior descending coronary artery; LCX, left circumflex coronary artery; LMCA, left main coronary artery; LVEF, left ventricular ejection fraction; ODT, onset-to-door time; PCI, percutaneous coronary intervention; RAAS, renin-angiotensin-aldosterone system; RCA, right coronary artery; SBP, systolic blood pressure; STEMI, ST-segment elevation myocardial infarction; TIMI, Thrombolysis in Myocardial Infarction.

As LVEF increased, the proportion of individuals with a body mass index (BMI) ≥25 kg/m^2^, dyslipidemia, and a family history of CAD increased, whereas those with Killip functional class III–IV, diabetes mellitus, multivessel disease, LMCA disease, femoral approach use, ACC/AHA lesion type B2/C, and an estimated glomerular filtration rate (eGFR) <60 mL/min/1.73 m^2^ decreased. Notably, both onset-to-door and door-to-balloon times showed significant intergroup differences, with patients in the snLVEF or preserved LVEF groups exhibiting slightly longer pre-hospital delays but shorter in-hospital reperfusion times compared to those with reduced LVEF. The proportions of individuals who underwent PCI, received glycoprotein IIb/IIIa inhibitors, underwent thrombus aspiration, had TIMI flow grade 0–I, and were diagnosed with STEMI were lowest in Group A. The completeness of PCI also differed significantly across groups, with the highest rate observed in Group B and the lowest in Group D (*P* < 0.001). Hemodynamic measurements at discharge revealed a stepwise decrease in both systolic and diastolic blood pressure (BP) as LVEF declined, whereas heart rate progressively increased with worsening LVEF. The incidence of in-hospital death increased progressively with decreasing LVEF, ranging from 0.6% in Group A to 8.3% in Group D (*P* < 0.001).

Despite differences in baseline risk, guideline-directed medical therapy was broadly implemented across the groups, with high overall use of aspirin, P2Y12 inhibitors, beta-blockers, renin-angiotensin-aldosterone system inhibitors, and statins, although statin prescription was numerically lower in Group D.

### 3.2 Clinical outcomes

**Figure 2:**
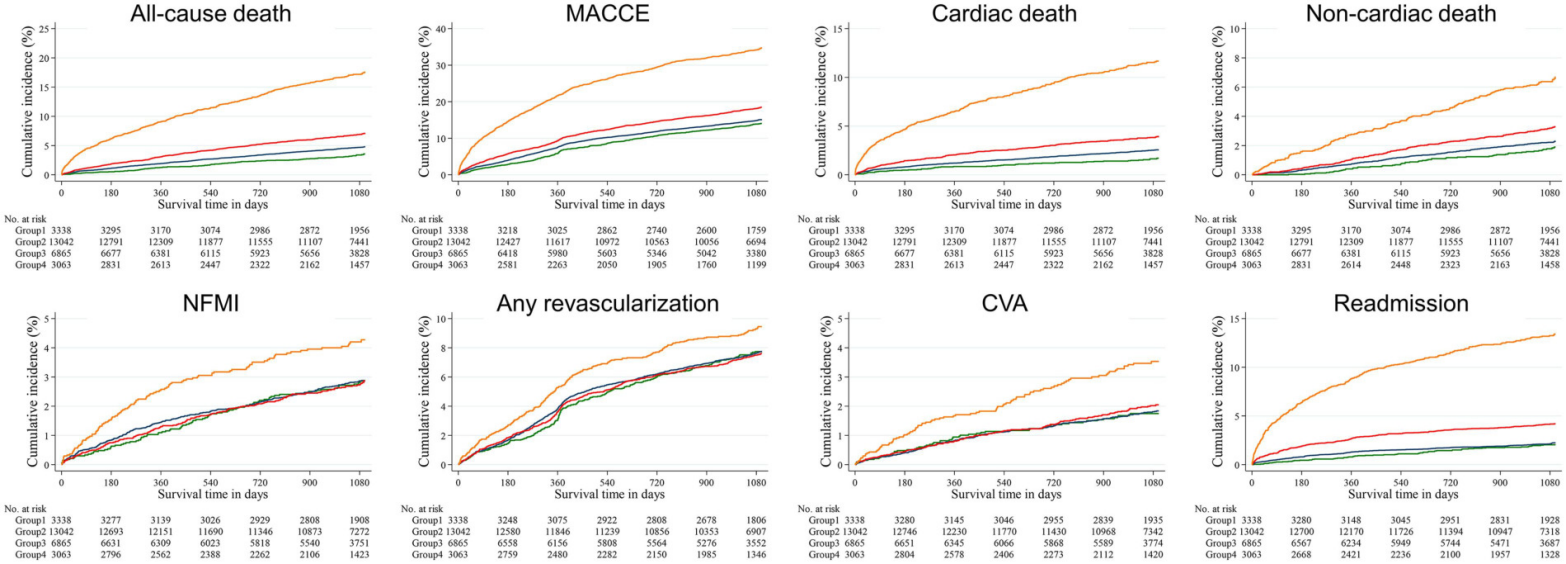
Kaplan–meier curves for clinical outcomes according to LVEF category. CVA, Cerebrovascular accident; LVEF, left ventricular ejection fraction; MACCE, major adverse cardiac and cerebrovascular accident; NFMI, non-fatal myocardial infarction.

Table 2 and Figure 2 summarize the crude incidence rates of all clinical outcomes and their associations with the LVEF. The median follow-up period was 1,088 days. Both the incidences and hazard ratios of most clinical outcomes, except NFMI, tended to decrease as the LVEF category increased. Across all four hierarchical Cox proportional hazards models (Models 1–4), patients with snLVEF (Group A) consistently exhibited the lowest incidence and hazard of all-cause death, cardiac death, and MACCE among the four groups. This trend persisted despite comprehensive adjustment for 35 covariates, including age, sex, comorbidities, coronary anatomy, procedural characteristics, and discharge medications, demonstrating a robust and consistent association between snLVEF and lower mortality in AMI. Log-minus-log survival plots did not suggest substantial deviation from the proportional hazards assumption across LVEF categories (Supplementary Figure S1).

**Table 2:** Associations between each group and clinical outcome with respect to each Cox model.

	Study group (LVEF category)	Events				
			Model 1 HR (95% CI)	Model 2 HR (95% CI)	Model 3 HR (95% CI)	Model 4 HR (95% CI)
All-cause death	Group A:	108 (3.2)	0.71 (0.58–0.87)	0.69 (0.56–0.84)	0.78 (0.61–0.99)	0.77 (0.60–0.98)
	LVEF ≥65%					
	Group B:	589 (4.5)	Reference			
	LVEF 50%–64%					
	Group C:	459 (6.7)	1.51 (1.33–1.70)	1.32 (1.17–1.49)	1.23 (1.06–1.42)	1.24 (1.06–1.44)
	LVEF 40%–49%					
	Group D:	510 (16.6)	4.10 (3.64–4.61)	2.94 (2.60–3.31)	2.04 (1.74–2.39)	1.92 (1.63–2.25)
	LVEF <40%					
Cardiac death	Group A:	52 (1.6)	0.64 (0.48–0.86)	0.62 (0.46–0.83)	0.70 (0.49–0.99)	0.70 (0.49–0.99)
	LVEF ≥65%					
	Group B:	313 (2.4)	Reference			
	LVEF 50%–64%					
	Group C:	252 (3.7)	1.55 (1.32–1.83)	1.36 (1.15–1.61)	1.22 (0.99–1.50)	1.24 (1.01–1.53)
	LVEF 40%–49%					
	Group D:	334 (10.9)	4.99 (4.28–5.82)	3.59 (3.07–4.19)	2.31 (1.88–2.83)	2.17 (1.76–2.68)
	LVEF <40%					
Non-cardiac death	Group A:	56 (1.7)	0.79 (0.59–1.05)	0.76 (0.57–1.01)	0.87 (0.62–1.21)	0.85 (0.61–1.19)
	LVEF ≥65%					
	Group B:	276 (2.1)	Reference			
	LVEF 50%–64%					
	Group C:	207 (3.0)	1.45 (1.21–1.74)	1.27 (1.06–1.52)	1.24 (0.99–1.54)	1.24 (0.99–1.55)
	LVEF 40%–49%					
	Group D:	176 (5.7)	3.06 (2.53–3.70)	2.19 (1.81–2.65)	1.68 (1.32–2.16)	1.58 (1.22–2.04)
	LVEF <40%					
MACCE	Group A:	447 (13.4)	0.91 (0.82–1.01)	0.90 (0.81–0.99)	0.94 (0.83–1.05)	0.96 (0.85–1.09)
	LVEF ≥65%					
	Group B:	1,890 (14.5)	Reference			
	LVEF 50%–64%					
	Group C:	1,211 (17.6)	1.25 (1.16–1.34)	1.18 (1.10–1.27)	1.09 (1.00–1.18)	1.10 (1.01–1.20)
	LVEF 40%–49%					
	Group D:	1,022 (33.4)	2.72 (2.52–2.94)	2.34 (2.16–2.52)	1.65 (1.50–1.82)	1.65 (1.49–1.82)
	LVEF <40%					
NFMI	Group A:	92 (2.8)	1.01 (0.80–1.27)	1.00 (0.79–1.26)	0.89 (0.67–1.18)	0.92 (0.69–1.23)
	LVEF ≥65%					
	Group B:	353 (2.7)	Reference			
	LVEF 50%–64%					
	Group C:	181 (2.6)	0.99 (0.83–1.18)	0.96 (0.80–1.15)	0.88 (0.71–1.08)	0.89 (0.71–1.10)
	LVEF 40%–49%					
	Group D:	115 (3.7)	1.54 (1.25–1.90)	1.41 (1.14–1.75)	0.97 (0.75–1.25)	0.99 (0.76–1.29)
	LVEF <40%					
Any revascularization	Group A:	243 (7.3)	0.99 (0.86–1.14)	0.99 (0.86–1.14)	1.04 (0.88–1.21)	1.07 (0.92–1.26)
	LVEF ≥65%					
	Group B:	947 (7.3)	Reference			
	LVEF 50%–64%					
	Group C:	482 (7.0)	0.98 (0.88–1.09)	0.98 (0.88–1.10)	0.92 (0.81–1.04)	0.95 (0.84–1.08)
	LVEF 40%–49%					
	Group D:	248 (8.1)	1.25 (1.08–1.43)	1.25 (1.08–1.44)	0.93 (0.79–1.10)	0.98 (0.83–1.17)
	LVEF <40%					
CVA	Group A:	55 (1.6)	0.94 (0.70–1.26)	0.92 (0.69–1.24)	1.01 (0.72–1.42)	1.04 (0.74–1.47)
	LVEF ≥65%					
	Group B:	227 (1.7)	Reference			
	LVEF 50%–64%					
	Group C:	126 (1.8)	1.07 (0.86–1.33)	1.01 (0.81–1.26)	0.96 (0.74–1.25)	0.98 (0.75–1.28)
	LVEF 40%–49%					
	Group D:	92 (3.0)	1.93 (1.52–2.46)	1.66 (1.30–2.12)	1.57 (1.16–2.11)	1.66 (1.22–2.27)
	LVEF <40%					
Readmission	Group A:	65 (1.9)	0.91 (0.70–1.20)	0.88 (0.67–1.15)	0.84 (0.60–1.17)	0.89 (0.64–1.25)
	LVEF ≥65%					
	Group B:	276 (2.1)	Reference			
	LVEF 50%–64%					
	Group C:	276 (4.0)	1.94 (1.64–2.30)	1.78 (1.50–2.10)	1.60 (1.31–1.95)	1.61 (1.31–1.98)
	LVEF 40%–49%					
	Group D:	372 (12.1)	6.57 (5.62–7.68)	5.26 (4.49–6.15)	3.76 (3.08–4.60)	3.74 (3.04–4.60)
	LVEF <40%					

CI, confidence interval; CVA, cerebrovascular accident; HR, hazard ratio; LVEF, left ventricular ejection fraction; MACCE, major adverse cardiac and cerebrovascular accident; NFMI, non-fatal myocardial infarction.

### 3.3 Restricted cubic spline analysis of LVEF and mortality

**Figure 3:**
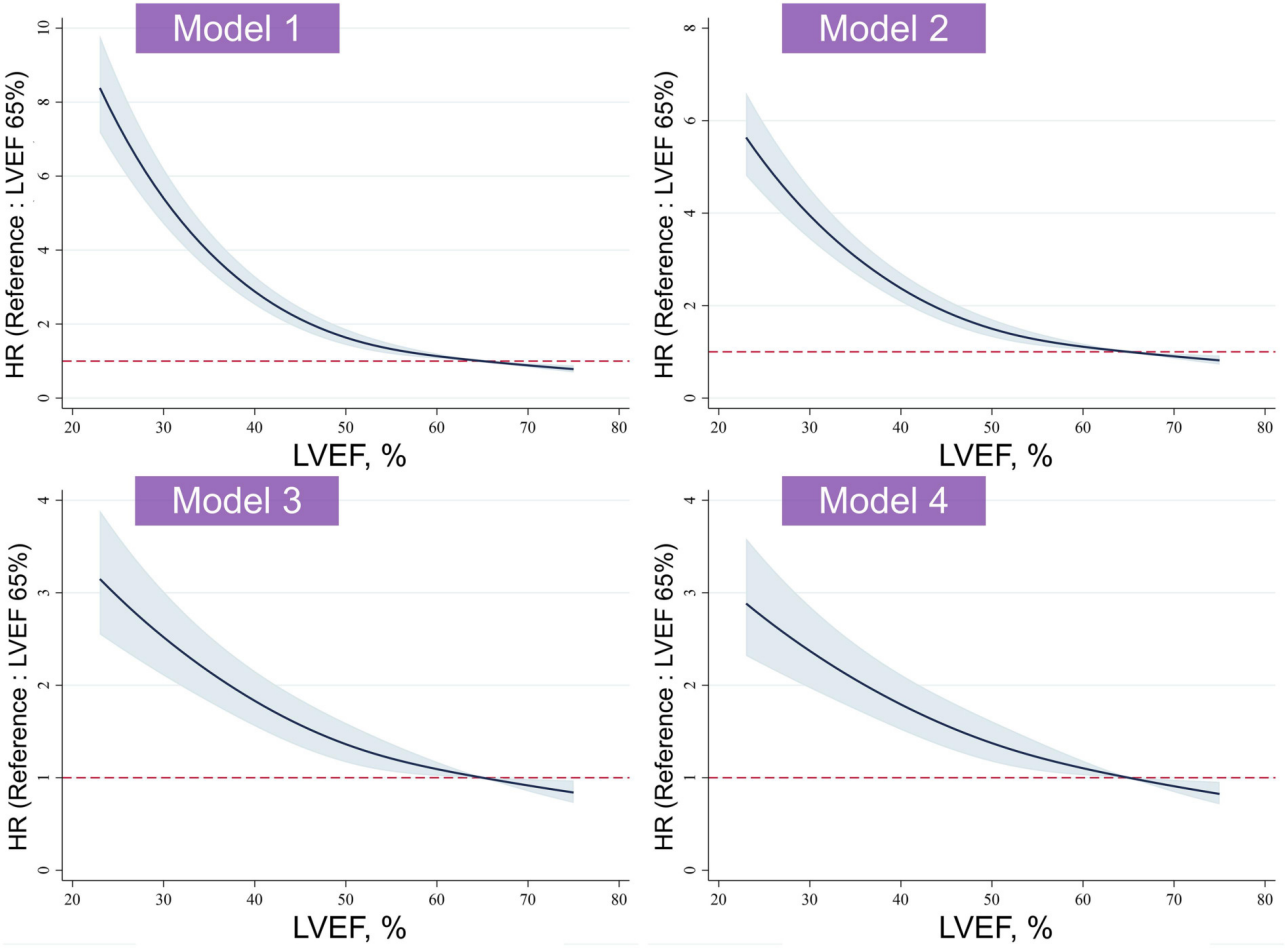
Restricted cubic spline analysis of LVEF and all-cause mortality. HR, hazard ratio; LVEF, left ventricular ejection fraction.

To evaluate the potential non-linear association between LVEF and all-cause mortality, we modeled LVEF as a continuous variable using restricted cubic splines within Cox proportional hazards regression models (Figure 3). Across Models 1–4, the spline curves demonstrated a monotonic inverse association between LVEF and mortality risk without evidence of a U-shaped pattern. The risk of mortality increased progressively as LVEF declined, with no excess mortality observed at higher LVEF ranges, including the supranormal range.

### 3.4 Sensitivity analysis using an alternative LVEF cutoff (≥60%)

To assess whether the findings were dependent on the predefined snLVEF cutoff of 65%, we performed a sensitivity analysis using an alternative threshold of ≥60% (Supplementary Tables S1 and S2). Across all four hierarchical models, patients with LVEF ≥60% remained associated with lower all-cause mortality compared with the reference group (LVEF 50%–59%), with effect sizes comparable to those observed in the primary analysis.

### 3.5 Subgroup-Specific analysis of All-cause death

To evaluate whether the survival advantage associated with snLVEF was consistent across clinically relevant strata, we conducted an exploratory subgroup analysis (Table 3). Overall, the direction of association consistently favored snLVEF in nearly all subgroups. The mortality risk reduction with snLVEF was particularly evident among patients aged <75 years (*P*-for-interaction <0.001), those without multivessel disease (*P*-for-interaction = 0.034), and those with preserved renal function (eGFR ≥ 60 mL/min/1.73 m^2^; *P*-for-interaction = 0.032). In subgroups defined by sex, hypertension, diabetes, and AMI type, the survival benefit of snLVEF remained directionally favorable without significant effect modification. These findings suggest that the prognostic impact of snLVEF on all-cause mortality was largely consistent across diverse clinical profiles but may be most pronounced in younger patients and those with single-vessel disease or intact renal function.

**Table 3:** Exploratory subgroup analysis comparing HRs for all-cause death according to LVEF category (fully-adjusted model).

	Study group (LVEF category)	Events	HR (95% CI)	*P*-value	Interaction *P*-value
Age					<0.001
**≥75 years**	Group A: LVEF ≥65%	65 (9.7)	0.75 (0.55–1.03)	0.073	
	Group B: LVEF 50%–64%	334 (13.5)	Reference		
	Group C: LVEF 40%–49%	257 (15.4)	1.07 (0.87–1.31)	0.520	
	Group D: VEF <40%	275 (27.2)	1.49 (1.20–1.86)	<0.001	
**<75 years**	Group A: LVEF ≥65%	43 (1.6)	0.80 (0.55–1.18)	0.264	
	Group B: LVEF 50%–64%	255 (2.4)	Reference		
	Group C: LVEF 40%–49%	202 (3.9)	1.54 (1.23–1.95)	<0.001	
	Group D: LVEF <40%	235 (11.4)	2.77 (2.16–3.54)	<0.001	
Sex					0.461
**Male**	Group A: LVEF ≥65%	63 (2.5)	0.79 (0.58–1.07)	0.131	
	Group B: LVEF 50%–64%	376 (3.7)	Reference		
	Group C: LVEF 40%–49%	283 (5.4)	1.26 (1.04–1.52)	0.020	
	Group D: LVEF <40%	330 (14.9)	2.06 (1.68–2.52)	<0.001	
**Female**	Group A: LVEF ≥65%	45 (5.3)	0.72 (0.48–1.08)	0.109	
	Group B: LVEF 50%–64%	213 (7.4)	Reference		
	Group C: LVEF 40%–49%	176 (10.5)	1.19 (0.92–1.54)	0.176	
	Group D:LVEF <40%	180 (21.2)	1.69 (1.29–2.23)	<0.001	
Hypertension					0.909
**Yes**	LVEF ≥65%	73 (4.2)	0.76 (0.56–1.03)	0.074	
	LVEF 50%–64%	381 (5.9)	Reference		
	LVEF 40%–49%	302 (9.0)	1.22 (1.01–1.47)	0.044	
	LVEF <40%	334 (20.2)	1.79 (1.46–2.20)	<0.001	
**No**	LVEF ≥65%	35 (2.2)	0.77 (0.51–1.18)	0.228	
	LVEF 50%–64%	208 (3.1)	Reference		
	LVEF 40%–49%	157 (4.4)	1.22 (0.95–1.57)	0.126	
	LVEF <40%	176 (12.5)	2.15 (1.64–2.82)	<0.001	
Diabetes mellitus					0.060
**Yes**	LVEF ≥65%	41 (5.1)	0.70 (0.47–1.05)	0.082	
	LVEF 50%–64%	258 (7.7)	Reference		
	LVEF 40%–49%	183 (9.6)	0.99 (0.78–1.27)	0.957	
	LVEF <40%	262 (21.9)	1.84 (1.45–2.34)	<0.001	
**No**	LVEF ≥65%	67 (2.6)	0.82 (0.60–1.11)	0.199	
	LVEF 50%–64%	331 (3.4)	Reference		
	LVEF 40%–49%	276 (5.6)	1.40 (1.15–1.70)	0.001	
	LVEF <40%	248 (13.3)	1.89 (1.52–2.36)	<0.001	
Multivessel disease					0.014
**Yes**	LVEF ≥65%	40 (2.8)	0.55 (0.38–0.80)	0.002	
	LVEF 50%–64%	310 (5.0)	Reference		
	LVEF 40%–49%	276 (7.8)	1.27 (1.04–1.54)	0.017	
	LVEF <40%	314 (16.4)	1.82 (1.48–2.23)	<0.001	
**No**	LVEF ≥65%	66 (3.5)	1.08 (0.78–1.50)	0.635	
	LVEF 50%–64%	258 (3.8)	Reference		
	LVEF 40%–49%	165 (5.0)	1.14 (0.90–1.46)	0.278	
	LVEF <40%	158 (14.6)	2.14 (1.65–2.78)	<0.001	
eGFR					0.045
**<60 mL/min/1.73 m^2^**	LVEF ≥65%	49 (12.4)	0.71 (0.48–1.03)	0.069	
	LVEF 50%–64%	252 (15.1)	Reference		
	LVEF 40%–49%	216 (17.7)	1.04 (0.82–1.32)	0.743	
	LVEF <40%	305 (29.1)	1.67 (1.33–2.11)	<0.001	
**≥60 mL/min/1.73 m^2^**	LVEF ≥65%	57 (1.9)	0.81 (0.59–1.12)	0.212	
	LVEF 50%–64%	336 (3.0)	Reference		
	LVEF 40%–49%	242 (4.3)	1.35 (1.11–1.65)	0.003	
	LVEF <40%	205 (10.2)	2.11 (1.68–2.66)	<0.001	
Final diagnosis					0.550
**STEMI**	LVEF ≥65%	27 (2.7)	0.96 (0.62–1.47)	0.840	
	LVEF 50%–64%	204 (3.5)	Reference		
	LVEF 40%–49%	198 (4.8)	1.10 (0.87–1.38)	0.440	
	LVEF <40%	184 (11.6)	1.76 (1.36–2.27)	<0.001	
**NSTEMI**	LVEF ≥65%	81 (3.5)	0.72 (0.53–0.97)	0.029	
	LVEF 50%–64%	385 (5.3)	Reference		
	LVEF 40%–49%	261 (9.6)	1.34 (1.09–1.65)	0.005	
	LVEF <40%	326 (22.1)	1.98 (1.59–2.45)	<0.001	

BMI, body mass index; CAD, coronary artery disease; CI, confidence interval; CVA, cerebrovascular accident; eGFR, estimated glomerular filtration rate; HR, hazard ratio; LVEF, left ventricular ejection fraction; MACCE, major adverse cardiac and cerebrovascular accident; NSTEMI, non-ST-segment elevation myocardial infarction; STEMI, ST-segment elevation myocardial infarction.

## 4 Discussion

This study evaluated the clinical outcomes of patients with AMI and snLVEF. In this nationwide cohort of patients with AMI, snLVEF was consistently associated with lower mortality compared with normal LVEF. snLVEF was consistently associated with lower mortality compared with normal LVEF in the AMI setting, and this association remained stable across hierarchical multivariable models.

Low LVEF, a major cause of mortality in patients with HF (7), is deemed one of the most important predictors of clinical outcomes among patients with AMI (2). However, a literature review revealed that limited data are available on the clinical characteristics of patients with snLVEF, a recently proposed concept. According to a *post-hoc* analysis of the RELAX-AHF-2 trial among patients with HF, patients with snLVEF had the best clinical outcomes, with the lowest risk of HF-related admission (8). Other studies have also demonstrated that subgroups with higher LVEF have a relatively low risk of HF-related admission (9, 10).

In the present study, we selected a cutoff value of 65% to evaluate AMI with snLVEF. This arbitrary cutoff value was based on clinical evidence from the literature review. In the EMPEROR-Preserved trial, the treatment effect of empagliflozin was attenuated among patients with LVEF ≥65% (10). According to data from the CONFIRM registry, a large observational cohort, patients with LVEF >65% had a higher mortality risk than those with lower LVEF (LVEF 55%–65% or LVEF <55%) (11). Other clinical studies also adopted this cutoff value to define snLVEF (8, 12). Importantly, our sensitivity analysis using an alternative cutoff of 60% yielded similar results, suggesting that the observed prognostic association is not dependent on a specific arbitrary threshold. Furthermore, restricted cubic spline modeling did not demonstrate a U-shaped relationship across the LVEF spectrum, supporting a graded inverse association between LVEF and mortality in the AMI setting.

Some notable findings regarding the baseline characteristics were identified. Patients with snLVEF had the highest proportion of BMI ≥ 25 kg/m^2^, but the lowest proportions of Killip class III–IV, diabetes mellitus, and eGFR < 60 mL/min/1.73 m^2^. Although still controversial, obesity is associated with favorable outcomes in patients with AMI (13). The Killip classification, a common tool for cardiovascular risk stratification, is related to clinical outcomes in patients with AMI (14). Moreover, diabetes mellitus and impaired kidney function are among the well-established risk factors for CAD (15, 16). Considering these aspects, all these factors may contribute directly or indirectly to better clinical outcomes in these patients. They also had the highest prevalence of dyslipidemia and a family history of CAD across all four groups. Considering that these two predisposing factors are among the well-established independent risk factors for CAD, including AMI, although not fully explainable, these findings suggest that these patients are aware of their risk factors. Furthermore, patients with snLVEF or preserved LVEF exhibited slightly longer onset-to-door time but shorter door-to-balloon time than those with reduced LVEF, implying that they generally presented with less hemodynamic compromise and required fewer stabilization procedures before revascularization, which may partly reflect differences in initial clinical severity and treatment urgency across LVEF categories.

Patients with snLVEF had the lowest proportions of multivessel and LMCA diseases. They also had the lowest proportions of ACC/AHA lesion type B2/C and TIMI flow grade 0–I. Based on these angiographic findings, these patients seemed to have a relatively low coronary atherosclerosis burden, thereby explaining the low incidence of STEMI. Therefore, they underwent the least aggressive revascularization treatment. In other words, the usage rates of PCI and thrombolysis were the lowest in this group, and they had the lowest rates of glycoprotein IIb/IIIa inhibitor use and thrombus aspiration.

Interestingly, patients with reduced LVEF exhibited lower systolic and diastolic BP and higher heart rates at discharge. These trends likely reflect impaired cardiac output and greater neurohormonal activation in those with more advanced left ventricular dysfunction. The inverse relationship between LVEF and BP, alongside the compensatory elevation in heart rate, highlights the hemodynamic burden and autonomic imbalance associated with systolic HF.

In the exploratory subgroup analyses, the mortality benefit associated with snLVEF was broadly consistent across most prespecified categories, with nominal heterogeneity observed according to age, coronary disease extent, and renal function. The apparent survival advantage of snLVEF was more pronounced among younger patients (<75 years), those without multivessel disease, and individuals with preserved renal function; however, these findings should be interpreted with caution because the multiple subgroup comparisons were performed without adjustment for multiplicity; thus, the possibility of chance associations cannot be excluded. Although subgroup patterns are hypothesis-generating rather than definitive, they suggest that the prognostic association of snLVEF may be somewhat more evident in younger patients with preserved renal function and less anatomically complex coronary disease.

To date, few studies have examined the prognostic implications of snLVEF in the context of acute coronary syndrome. Li et al. previously reported that patients with acute coronary syndrome and snLVEF exhibited worse outcomes compared to those with normal LVEF (17). However, several important methodological differences should be considered when interpreting the apparent discrepancy between their findings and ours. First, their study population consisted of a heterogeneous acute coronary syndrome cohort, with a very small proportion of patients with confirmed AMI in the snLVEF category, which may have limited statistical stability and disease-specific interpretation. Second, differences in outcome definitions and follow-up duration may have influenced risk estimates. Third, their multivariable adjustment strategy did not comprehensively account for angiographic complexity, infarct-related artery characteristics, procedural variables, and discharge medical therapy, whereas our hierarchical modeling framework incorporated detailed clinical, angiographic, and procedural covariates (Supplementary Data S1). These methodological distinctions suggest that the divergent findings may reflect differences in study design and population characteristics rather than indicating a fundamentally different biological relationship.

snLVEF is often regarded as a normal LVEF, and patients with snLVEF are generally considered to have an excellent prognosis. However, evidence regarding the U-shaped relationship between LVEF and mortality has been accumulating (18). In a 4-year follow-up observational study conducted by Wehner et al. (19), as mortality rates increased with a reduction in LVEF, its rates also increased among patients with snLVEF. Ohte et al. reported similar results for acute decompensated HF (20), suggesting that snLVEF may be a risk factor in this setting. According to a clinical study based on data from the Global Registry of Acute Coronary Events, snLVEF appeared to be associated with higher rates of in-hospital mortality among patients with established acute coronary syndrome compared to those with normal LVEF (21). A clinical study based on the RELAX-AHF-2 trial reported that patients with snLVEF showed a higher rate of non-cardiac death than those with normal LVEF, despite the comparable incidence of cardiac death and even lower incidence of rehospitalization (8). This was also observed for acute or chronic HF (22, 23), even among community-dwelling adults without established cardiovascular disorders (24).

Unlike the U-shaped relationship reported in heart failure and community-based cohorts, our analysis demonstrated a graded inverse association between LVEF and mortality in the AMI setting. Given that LVEF in the acute phase of myocardial infarction may be closely linked to infarct size (25–27), the favorable prognosis observed in patients with snLVEF may, at least in part, reflect smaller myocardial injury rather than an independent biological effect of high LVEF *per se*. Indeed, biochemical markers of necrosis and markers of ventricular remodeling were lowest and most favorable in the supranormal group (Supplementary Table S3), supporting the interpretation that these patients experienced relatively limited myocardial damage.

However, LVEF measured during index hospitalization may represent more than infarct size alone. In parallel, echocardiographic indices—including wall motion score index and left ventricular end-systolic and end-diastolic diameters—were most favorable in this group, indicating preserved chamber geometry (Supplementary Table S3). Since left ventricular dilatation after AMI is a hallmark of adverse remodeling and a well-established predictor of subsequent HF and mortality (28, 29), the smaller left ventricular dimensions observed in the snLVEF group support the interpretation that reduced remodeling underlie their superior outcomes.

Beyond infarct size and remodeling, snLVEF in the acute phase may also reflect transient hyperdynamic physiology driven by heightened sympathetic activation or altered loading conditions (30). In certain contexts, such hypercontractile states have been associated with adverse outcomes, particularly in chronic heart failure or concentric remodeling phenotypes (31–33). Additionally, because LVEF is load-dependent and operator-measured, measurement variability and differences in ventricular geometry may contribute to apparent supranormal values. However, in our AMI cohort, patients with snLVEF did not exhibit features suggestive of adverse hyperdynamic states; rather, they demonstrated lower inflammatory burden, less neurohormonal activation, and more favorable ventricular dimensions, supporting the interpretation that snLVEF in this setting predominantly represents a low-risk post-infarction phenotype.

To our knowledge, this study is the first to demonstrate the clinical characteristics and outcomes of patients with AMI and snLVEF. It used a well-established nationwide AMI cohort in which the LVEF was assessed at the time of index hospitalization. Our novel investigation highlights a negative correlation between LVEF and clinical outcomes in the AMI setting, instead of the U-shaped relationship shown in the aforementioned studies.

However, this study had inherent limitations. First, as in all observational cohort-based studies, the clinical outcomes may have been influenced by confounders. Despite our efforts to adjust for covariates using Cox proportional hazards regression models, potential selection bias may have persisted owing to the inclusion and exclusion criteria, the exclusion of data with missing values, and the possibility of residual or unmeasured confounders. In particular, we did not include certain important clinical variables—such as other comorbidities (e.g., atrial fibrillation, or chronic kidney disease)—which may partly explain the large magnitude of risk reduction attributed to patients with snLVEF. Second, as LVEF quantification was performed manually at the discretion of the investigator rather than performed by a core echocardiography laboratory, measurement variability and misclassification may have occurred across LVEF strata. Third, we did not conduct a competing risk analysis, which could have influenced risk estimates, particularly in the context of non-cardiac vs. cardiac mortality. In addition, although our multivariable models already incorporated key indicators of disease extent—such as STEMI presentation, multivessel disease, and lesion complexity—we did not perform an additional sensitivity analysis specifically adjusting for these factors. In our subgroup analyses, the possibility of residual confounding is further amplified, which necessitates cautious interpretation of statistical significance, owing to the increased likelihood of type I error from multiple comparisons. Fourth, since the present analysis was based exclusively on a Korean nationwide cohort, population-specific factors—including ethnic differences in cardiovascular risk profiles, treatment strategies, and genetic predisposition—may limit the direct generalizability of our findings to non-Asian populations. Finally, as this was a *post-hoc* retrospective analysis, our results should be considered hypothesis-generating, and future prospective, multinational investigations with standardized echocardiographic assessment are warranted to validate and expand these observations.

From a clinical perspective, our findings suggest that snLVEF in the setting of AMI should not be interpreted as a marker of increased mortality risk, in contrast to prior reports in heart failure or community-based populations. Rather, snLVEF appears to be associated with a favorable risk profile in this context. However, this should not lead to complacency in clinical management. Risk stratification in AMI should remain multifactorial, incorporating clinical presentation, infarct size, renal function, coronary anatomy, and guideline-directed medical therapy. The presence of snLVEF alone should not alter established secondary prevention strategies but may provide reassurance regarding short- and mid-term prognosis within a comprehensive clinical framework.

In conclusion, patients with snLVEF appeared to have more favorable clinical outcomes than those with lower LVEF in the AMI setting. These findings should be interpreted with caution and considered hypothesis-generating. Further prospective studies are warranted to validate and extend these observations.

## Data Availability

The data generated and/or analyzed during the current study are available from the corresponding authors upon reasonable request.

## Supplementary material

Supplementary Figure S1 Log-minus-log survival plots according to LVEF category Log-minus-log survival curves for all-cause mortality according to LVEF category (Group A–D). The approximate parallelism of the curves supports the proportional hazards assumption for the Cox regression models. LVEF, left ventricular ejection fraction.

Supplementary Table S1 Baseline characteristics of study participants according to LVEF category (Group A–D). Values are presented as mean ± standard deviation or number (percentage).

Supplementary Table S2 Associations between LVEF category and clinical outcomes across four hierarchical Cox proportional hazards models.

Supplementary Table S3 Biochemical markers and additional echocardiographic parameters according to LVEF category.
