# Supplementary figures and images for: Outcomes for acute myocardial infarction with supranormal left ventricular ejection fraction
Source: Front Cardiovasc Med. 2026 Apr 10;13:1777247. doi: 10.3389/fcvm.2026.1777247 (PMC13106417; doi:10.3389/fcvm.2026.1777247)

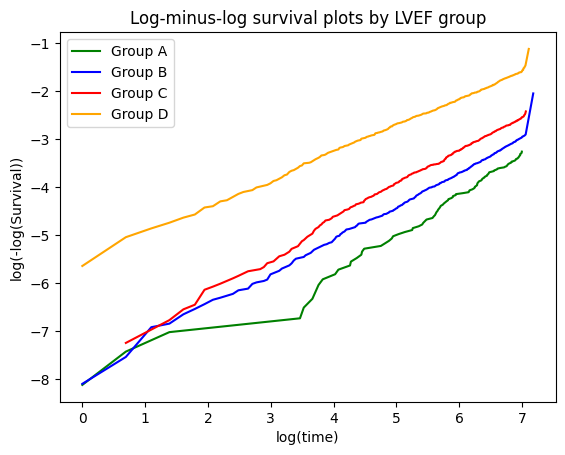

Supplement: Supplementary Figure S1 — Log-minus-log survival plots according to LVEF category Log-minus-log survival curves for all-cause mortality according to LVEF category (Group A–D). The approximate parallelism of the curves supports the proportional hazards assumption for the Cox regression models. LVEF, left ventricular ejection fraction. [file Image1.tif]
