# Supplementary material for: Outcomes for acute myocardial infarction with supranormal left ventricular ejection fraction
Source: Front Cardiovasc Med. 2026 Apr 10;13:1777247. doi: 10.3389/fcvm.2026.1777247 (PMC13106417; doi:10.3389/fcvm.2026.1777247)
Supplement: Supplementary Table S1 — Baseline characteristics of study participants according to LVEF category (Group A–D). Values are presented as mean ± standard deviation or number (percentage). [file Table1.docx]

**Supplemental Table S1**. Baseline characteristics of study participants

|  | **Group A** LVEF ≥60%  **(n=7325)** | **Group B** LVEF 50–59%  **(n=9657)** | **Group C** LVEF 40–49%  **(n=7315)** | **Group D** LVEF <40%  **(n=3606)** | ***P*-value  for trend** |
| --- | --- | --- | --- | --- | --- |
| Age, years | 62.72±12.16 | 62.86±12.38 | 64.56±12.67 | 68.30±12.40 | <0.001 |
| Age ≥75 years | 1421 (19.4) | 1956 (20.2) | 1882 (25.7) | 1288 (35.7) | <0.001 |
| Male sex | 5546 (75.7) | 7553 (78.2) | 5477 (74.9) | 2587 (71.7) | <0.001 |
| ODT, h | 35.34±218.16 | 27.77±222.35 | 26.45±180.45 | 38.70±200.95 | <0.001 |
| DBT, h | 16.08±34.82 | 12.98±33.17 | 11.23±34.37 | 19.83±57.98 | <0.001 |
| BMI ≥25 kg/m^2^ | 2850 (40.9) | 3496 (38.4) | 2384 (34.6) | 946 (28.1) | <0.001 |
| Killip class III-IV | 400 (5.6) | 745 (7.8) | 851 (11.8) | 1051 (29.7) | <0.001 |
| Comorbidities |  |  |  |  |  |
| Hypertension | 3674 (50.2) | 4780 (49.5) | 3595 (49.2) | 1989 (55.2) | 0.001 |
| Diabetes mellitus | 1785 (24.4) | 2548 (26.4) | 2061 (28.2) | 1464 (40.6) | <0.001 |
| Dyslipidemia | 1064 (14.5) | 1238 (12.8) | 878 (12.0) | 423 (11.7) | <0.001 |
| Prior CAD | 1052 (14.4) | 1251 (12.9) | 1062 (14.5) | 799 (22.2) | <0.001 |
| Prior CVA | 439 (6.0) | 541 (5.6) | 513 (7.0) | 378 (10.5) | <0.001 |
| Smoking history | 4144 (58.2) | 5564 (59.3) | 4053 (56.9) | 1790 (51.5) | <0.001 |
| Family history of CAD | 611 (8.6) | 764 (8.1) | 447 (6.3) | 205 (5.9) | <0.001 |
| Use of thrombolysis | 35 (0.5) | 76 (0.8) | 92 (1.3) | 22 (0.6) | 0.004 |
| Multivessel disease | 3292 (45.2) | 4688 (48.9) | 3776 (52.0) | 2278 (64.8) | <0.001 |
| LMCA disease | 307 (4.2) | 395 (4.1) | 343 (4.7) | 320 (9.1) | <0.001 |
| Use of PCI | 6502 (88.8) | 8983 (93.0) | 6889 (94.2) | 3187 (88.4) | <0.001 |
| Use of femoral approach | 2892 (44.5) | 4696 (52.3) | 3883 (56.4) | 2001 (62.8) | <0.001 |
| Use of GPIIb/IIIa inhibitors | 627 (8.6) | 1244 (12.9) | 1031 (14.1) | 376 (10.4) | <0.001 |
| Use of thrombus aspiration | 861 (11.7) | 1810 (18.7) | 1508 (20.6) | 592 (16.4) | <0.001 |
| Use of intracoronary imaging | 1718 (23.4) | 2246 (23.3) | 1701 (23.3) | 640 (17.7) | <0.001 |
| Infarct-related artery |  |  |  |  | <0.001 |
| LMCA or LAD | 2785 (42.8) | 3566 (39.7) | 4122 (59.8) | 2095 (65.7) |  |
| LCX or RCA | 3726 (57.2) | 5423 (60.3) | 2770 (40.2) | 1092 (34.3) |  |
| ACC/AHA lesion type B2/C | 5207 (82.6) | 7530 (86.3) | 5729 (86.5) | 2738 (89.0) | <0.001 |
| TIMI flow grade 0-I | 2958 (46.1) | 5257 (59.1) | 4612 (67.8) | 1942 (62.0) | <0.001 |
| LVEF, % | 65.14±4.43 | 54.75±2.88 | 45.24±2.75 | 31.97±6.22 | <0.001 |
| eGFR <60 mL/min/1.73m^2^ | 900 (12.3) | 1330 (13.8) | 1378 (18.9) | 1361 (37.8) | <0.001 |
| STEMI as a final diagnosis | 2431 (33.2) | 4562 (47.2) | 4415 (60.4) | 1879 (52.1) | <0.001 |
| SBP at discharge, mmHg | 117.26±15.32 | 115.30±15.29 | 112.99±15.18 | 111.56±16.05 | <0.001 |
| DBP at discharge, mmHg | 70.07±10.14 | 69.04±10.17 | 68.04±10.06 | 66.70±10.12 | <0.001 |
| Heart rate at discharge, bpm | 69.20±10.01 | 70.31±10.42 | 72.32±10.68 | 75.23±12.10 | <0.001 |
| Medications |  |  |  |  |  |
| Aspirin | 7260 (99.1) | 9611 (99.5) | 7279 (99.5) | 3557 (98.6) | 0.181 |
| P2Y12 inhibitors | 7237 (98.8) | 9585 (99.3) | 7258 (99.2) | 3547 (98.4) | 0.334 |
| Beta-blockers | 5579 (76.2) | 7789 (80.7) | 5951 (81.4) | 2565 (71.1) | 0.031 |
| RAAS inhibitors | 5553 (75.8) | 7389 (76.5) | 5524 (75.5) | 2450 (67.9) | <0.001 |
| Statins | 6920 (94.5) | 9127 (94.5) | 6829 (93.4) | 3046 (84.5) | <0.001 |

ACC/AHA, American College of Cardiology/American Heart Association; BMI, body mass index; CAD, coronary artery disease; CVA, cerebrovascular accident; DBP, diastolic blood pressure; DBT, door-to-balloon time; eGFR, estimated glomerular filtration rate; GPIIb/IIIa, glycoprotein IIb/IIIa; LAD, left anterior descending coronary artery; LCX, left circumflex coronary artery; LMCA, left main coronary artery; LVEF, left ventricular ejection fraction; ODT, onset-to-door time; PCI, percutaneous coronary intervention; RAAS, renin-angiotensin-aldosterone system; RCA, right coronary artery; SBP, systolic blood pressure; STEMI, ST-segment elevation myocardial infarction; TIMI, Thrombolysis in Myocardial Infarction
