# Supplementary material for: Outcomes for acute myocardial infarction with supranormal left ventricular ejection fraction
Source: Front Cardiovasc Med. 2026 Apr 10;13:1777247. doi: 10.3389/fcvm.2026.1777247 (PMC13106417; doi:10.3389/fcvm.2026.1777247)
Supplement: Supplementary Table S2 — Associations between LVEF category and clinical outcomes across four hierarchical Cox proportional hazards models. [file Table2.docx]

**Supplemental Table S2**. Associations between each group and clinical outcome with respect to each Cox model

|  |  |  | **HR (95% CI)** | | | |
| --- | --- | --- | --- | --- | --- | --- |
|  | **Study group  (LVEF category)** | **Events** | **Model 1** | **Model 2** | **Model 3** | **Model 4** |
| **All-cause death** | Group A: | 236 (3.3) | 0.67 (0.57-0.78) | 0.68 (0.58-0.79) | 0.76 (0.63-0.92) | 0.77 (0.64-0.93) |
|  | LVEF ≥60% |  |  |  |  |  |
|  | Group B: | 461 (4.9) | Reference | | | |
|  | LVEF 50–59% |  |  |  |  |  |
|  | Group C: | 459 (6.7) | 1.37 (1.20-1.56) | 1.22 (1.07-1.38) | 1.16 (0.99-1.36) | 1.18 (1.00-1.38) |
|  | LVEF 40–49% |  |  |  |  |  |
|  | Group D: | 510 (16.6) | 3.73 (3.29-4.23) | 2.71 (2.39-3.08) | 1.93 (1.64-2.28) | 1.83 (1.54-2.16) |
|  | LVEF <40% |  |  |  |  |  |
| **Cardiac death** | Group A: | 114 (1.6) | 0.59 (0.48-0.74) | 0.60 (0.48-0.75) | 0.65 (0.50-0.85) | 0.66 (0.50-0.87) |
|  | LVEF ≥60% |  |  |  |  |  |
|  | Group B: | 251 (2.7) | Reference | | | |
|  | LVEF 50–59% |  |  |  |  |  |
|  | Group C: | 252 (3.7) | 1.38 (1.16-1.64) | 1.22 (1.03-1.46) | 1.12 (0.90-1.38) | 1.14 (0.92-1.42) |
|  | LVEF 40–49% |  |  |  |  |  |
|  | Group D: | 334 (10.9) | 4.43 (3.76-5.22) | 3.23 (2.74-3.81) | 2.11 (1.71-2.62) | 2.00 (1.60-2.49) |
|  | LVEF <40% |  |  |  |  |  |
| **Non-cardiac death** | Group A: | 122 (1.7) | 0.76 (0.61-0.95) | 0.77 (0.62-0.96) | 0.90 (0.69-1.18) | 0.90 (0.69-1.18) |
|  | LVEF ≥60% |  |  |  |  |  |
|  | Group B: | 210 (2.3) | Reference | | | |
|  | LVEF 50–59% |  |  |  |  |  |
|  | Group C: | 207 (3.0) | 1.36 (1.12-1.65) | 1.21 (1.00-1.47) | 1.22 (0.96-1.54) | 1.22 (0.96-1.56) |
|  | LVEF 40–49% |  |  |  |  |  |
|  | Group D: | 176 (5.7) | 2.87 (2.35-3.50) | 2.08 (1.70-2.55) | 1.66 (1.28-2.16) | 1.56 (1.20-2.05) |
|  | LVEF <40% |  |  |  |  |  |
| **MACCE** | Group A: | 957 (13.5) | 0.90 (0.83-0.98) | 0.90 (0.83-0.98) | 0.97 (0.88-1.06) | 0.99 (0.90-1.10) |
|  | LVEF ≥60% |  |  |  |  |  |
|  | Group B: | 1380 (14.8) | Reference | | | |
|  | LVEF 50–59% |  |  |  |  |  |
|  | Group C: | 1211 (17.6) | 1.22 (1.13-1.31) | 1.16 (1.07-1.25) | 1.09 (0.99-1.19) | 1.11 (1.01-1.21) |
|  | LVEF 40–49% |  |  |  |  |  |
|  | Group D: | 1022 (33.4) | 2.66 (2.45-2.88) | 2.29 (2.11-2.48) | 1.65 (1.49-1.82) | 1.66 (1.49-1.84) |
|  | LVEF <40% |  |  |  |  |  |
| **NFMI** | Group A: | 198 (2.8) | 1.05 (0.87-1.26) | 1.05 (0.87-1.26) | 1.01 (0.81-1.26) | 1.04 (0.83-1.31) |
|  | LVEF ≥60% |  |  |  |  |  |
|  | Group B: | 247 (2.6) | Reference | | | |
|  | LVEF 50–59% |  |  |  |  |  |
|  | Group C: | 181 (2.6) | 1.01 (0.83-1.22) | 0.98 (0.81-1.18) | 0.90 (0.72-1.12) | 0.91 (0.73-1.15) |
|  | LVEF 40–49% |  |  |  |  |  |
|  | Group D: | 115 (3.7) | 1.57 (1.26-1.96) | 1.44 (1.15-1.80) | 0.99 (0.76-1.30) | 1.02 (0.77-1.34) |
|  | LVEF <40% |  |  |  |  |  |
| **Any revascularization** | Group A: | 536 (7.6) | 1.07 (0.96-1.20) | 1.07 (0.96-1.20) | 1.15 (1.01-1.30) | 1.18 (1.04-1.35) |
|  | LVEF ≥60% |  |  |  |  |  |
|  | Group B: | 654 (7.0) | Reference | | | |
|  | LVEF 50–59% |  |  |  |  |  |
|  | Group C: | 482 (7.0) | 1.01 (0.90-1.14) | 1.01 (0.90-1.14) | 0.97 (0.85-1.10) | 1.00 (0.88-1.15) |
|  | LVEF 40–49% |  |  |  |  |  |
|  | Group D: | 248 (8.1) | 1.29 (1.11-1.49) | 1.29 (1.11-1.49) | 0.98 (0.83-1.16) | 1.04 (0.87-1.24) |
|  | LVEF <40% |  |  |  |  |  |
| **CVA** | Group A: | 107 (1.5) | 0.80 (0.63-1.01) | 0.79 (0.62-1.01) | 0.73 (0.55-0.98) | 0.76 (0.57-1.01) |
|  | LVEF ≥60% |  |  |  |  |  |
|  | Group B: | 175 (1.9) | Reference | | | |
|  | LVEF 50–59% |  |  |  |  |  |
|  | Group C: | 126 (1.8) | 0.99 (0.79-1.24) | 0.94 (0.74-1.18) | 0.86 (0.66-1.12) | 0.87 (0.66-1.15) |
|  | LVEF 40–49% |  |  |  |  |  |
|  | Group D: | 92 (3.0) | 1.78 (1.39-2.30) | 1.53 (1.19-1.98) | 1.40 (1.03-1.90) | 1.49 (1.09-2.05) |
|  | LVEF <40% |  |  |  |  |  |
| **Readmission** | Group A: | 136 (1.9) | 0.86 (0.70-1.07) | 0.86 (0.69-1.06) | 0.88 (0.68-1.14) | 0.92 (0.71-1.20) |
|  | LVEF ≥60% |  |  |  |  |  |
|  | Group B: | 205 (2.2) | Reference | | | |
|  | LVEF 50–59% |  |  |  |  |  |
|  | Group C: | 276 (4.0) | 1.86 (1.55-2.23) | 1.71 (1.43-2.05) | 1.57 (1.27-1.94) | 1.60 (1.28-1.99) |
|  | LVEF 40–49% |  |  |  |  |  |
|  | Group D: | 372 (12.1) | 6.29 (5.31-7.46) | 5.06 (4.26-6.00) | 3.69 (2.98-4.58) | 3.69 (2.95-4.62) |
|  | LVEF <40% |  |  |  |  |  |

CI, confidence interval; CVA, cerebrovascular accident; HR, hazard ratio; LVEF, left ventricular ejection fraction; MACCE, major adverse cardiac and cerebrovascular accident; NFMI, non-fatal myocardial infarction.
