# Supplementary material for: Outcomes for acute myocardial infarction with supranormal left ventricular ejection fraction
Source: Front Cardiovasc Med. 2026 Apr 10;13:1777247. doi: 10.3389/fcvm.2026.1777247 (PMC13106417; doi:10.3389/fcvm.2026.1777247)
Supplement: Supplementary Table S3 — Biochemical markers and additional echocardiographic parameters according to LVEF category. [file Table3.docx]

**Supplementary Table S3**. Biochemical markers and other echocardiographic parameters

|  | Group A | Group B | Group C | Group D | *P*-value  for trend |
| --- | --- | --- | --- | --- | --- |
| Biochemical markers |  |  |  |  |  |
| NT-proBNP, pg/mL | 917.32±2933.16 | 1272.81±4196.13 | 2741.51±6877.06 | 7853.02±13428.01 | <0.001 |
| Initial CK-MB, mg/dL | 50.96±88.13 | 91.46±113.59 | 154.52±170.48 | 139.26±210.23 | <0.001 |
| Initial troponin-I, mg/dL | 15.87±37.44 | 34.50±76.30 | 61.50±105.17 | 65.55±115.84 | <0.001 |
| hs-CRP, mg/dL | 0.73±2.00 | 1.04±3.31 | 1.69±5.56 | 3.22±7.41 | <0.001 |
| Echocardiographic parameters |  |  |  |  |  |
| WMSI | 1.07±0.39 | 1.26±1.11 | 1.64±0.35 | 1.97±0.46 | <0.001 |
| LVESD, mm | 28.60±5.11 | 32.38±6.62 | 36.02±8.53 | 42.99±11.13 | <0.001 |
| LVESD ≥45mm, % | 17 (0.5) | 199 (1.6) | 643 (9.6) | 1405 (44.2) | <0.001 |
| LVEDD, mm | 47.58±5.80 | 48.82±6.46 | 50.61±6.87 | 54.47±9.85 | <0.001 |
| LVEDD ≥55mm, % | 204 (6.4) | 1362 (10.9) | 1506 (22.3) | 1566 (48.5) | <0.001 |

CK-MB, creatine kinase-MB; hs-CRP, high-sensitivity C-reactive protein; LVEDD, left ventricular end-diastolic diameter; LVESD, left ventricular end-systolic diameter; NT-proBNP, N-terminal pro-B-type natriuretic peptide; WMSI, wall motion score index.
