# Supplementary material for: Outcomes for acute myocardial infarction with supranormal left ventricular ejection fraction
Source: Front Cardiovasc Med. 2026 Apr 10;13:1777247. doi: 10.3389/fcvm.2026.1777247 (PMC13106417; doi:10.3389/fcvm.2026.1777247)
Supplement: Supplementary file 5 [file Supplementaryfile1.docx]

**Supplementary Data S1.** Detailed Information on the Cox Proportional Hazards Regression Models

To comprehensively evaluate the association between left ventricular ejection fraction (LVEF) category and clinical outcomes, we constructed four hierarchical Cox proportional hazards regression models with incremental adjustment for potential confounders. Covariates were selected a priori based on clinical relevance, biological plausibility, and availability within the registry dataset.

1. **Model 1: Unadjusted Model**

Model 1 was a crude model including only the LVEF category (groups A-D) as the independent variable, without adjustment for any covariates.

1. **Model 2: Age- and Sex-Adjusted Model**

Model 2 was adjusted for the two fundamental demographic variables: Age (continuous) Sex (male vs. female)

1. **Model 3: Clinical and Angiographic Adjusted Model**

Model 3 was additionally adjusted for baseline clinical characteristics, comorbidities, and angiographic/procedural variables that may influence prognosis after acute myocardial infarction (AMI), excluding discharge medications and discharge hemodynamic parameters.

The following covariates were included:

**Demographic and Time Variables**

- Age
- Sex (male vs. female)
- Onset-to-door time
- Door-to-balloon time

**Clinical Characteristics**

- Body mass index (≥25 vs. <25 kg/m²)
- Killip functional class (I–II vs. III–IV)
- Hypertension
- Diabetes mellitus
- Dyslipidemia
- Prior coronary artery disease (CAD)
- Prior cerebrovascular accident
- Smoking history
- Family history of CAD

**Renal Function**

- Estimated glomerular filtration rate (<60 vs. ≥60 mL/min/1.73 m²)

**Coronary Anatomy and Lesion Characteristics**

- Multivessel disease
- Left main coronary artery (LMCA) disease
- Infarct-related artery (LMCA or left anterior descending coronary artery vs. left circumflex coronary artery or right coronary artery)
- American College of Cardiology/American Heart Association (ACC/AHA) lesion type (ACC/AHA type A/B1 vs. ACC/AHA type B2/C)
- Thrombolysis In Myocardial Infarction (TIMI) flow grade (TIMI 0–I vs. TIMI II–III)

**Procedural Variables**

- Application of complete percutaneous coronary intervention (PCI)
  (complete PCI vs. incomplete PCI)
- Vascular approach (femoral vs. non-femoral)
- Use of glycoprotein IIb/IIIa inhibitors
- Use of thrombus aspiration
- Use of intracoronary imaging

**Final Diagnosis**

- Type of AMI (ST-segment elevation myocardial infarction vs. non-ST-segment elevation myocardial infarction)

1. **Model 4: Fully Adjusted Model**

Model 4 was the fully adjusted model and included all covariates from Model 3 plus discharge hemodynamic parameters and discharge medications.

### Additional Hemodynamic Parameters

- Systolic blood pressure at discharge
- Diastolic blood pressure at discharge
- Heart rate at discharge

### Discharge Medications

- Aspirin
- P2Y12 inhibitors
- Beta-blockers
- Renin–angiotensin–aldosterone system inhibitors
- Statins

1. **Model 4: Fully Adjusted Model**

- Hazard ratios and 95% confidence intervals were estimated using Cox proportional hazards regression.
- The proportional hazards assumption was assessed using Schoenfeld residuals.
- Patients with missing values for any covariates included in each model were excluded from that specific analysis (complete-case approach).
- Covariates were selected based on prespecified clinical relevance rather than automated selection procedures.
